# Supplementary material for: Metalation of a Hierarchical Self-Assembly Consisting of π-Stacked Cubes through Single-Crystal-to-Single-Crystal Transformation
Source: Molecules. 2023 Jun 22;28(13):4923. doi: 10.3390/molecules28134923 (PMC10343287; doi:10.3390/molecules28134923)
Supplement: Supplementary file 1 [file molecules-28-04923-s001.zip › molecules-2458815-supplementary.pdf]

Metalation of a Hierarchical Self-Assembly Consisting π-Stacked Cubes through Single-Crystal to Single-Crystal Transformation

Table S1. Crystallographic data of the synthetic compounds (1 and 2).

Compounds	**1**	**2**
Formula	$C_{216}H_{304}N_{59}Cl_{15}O_{92}$	$C_{235}H_{254}Co_{8}N_{78}S_{19}O_{13}$
Formula weight	5720.62	5455.09
Temp. (*K*)	200	200
Crystal System	Cubic	Cubic
Space group	$Fm\bar{3}c$	$Fm\bar{3}c$
a (Å)	39.9644(11)	38.6558(16)
b (Å)	39.9644(11)	38.6558(16)
c (Å)	39.9644(11)	38.6558(16)
a (°)	90	90
β (°)	90	90
γ (°)	90	90
V(Å^3)	63829(5)	57762(7)
Z	8	8
$\rho_{cal.}$(g cm^{-3})	1.204	1.363
μ	0.214	0.663
F(000)	24264.0	24752.0
θ *range* (°)	4.076–42.158	4.214–49.982
Reflections ($I > 2\sigma$)	1044	2383
R_{int}	0.0407	0.0400
R_{sigma}	0.0167	0.0051
Data/restraints/parameters	1540/167/253	2231/42/192
$R_1(I > 2\sigma)$	0.1907	0.1178
$wR_2(all)$	0.5038	0.4362
GOF on F^2	2.259	2.247
CCDC#	2266971	2266972

[a] $R = \sum||F_0|-|F_c||)/\sum|F_0|$

[b] $wR = |\sum w(F_0 - F_c)^2 / \sum w(F_0^2)^2|^{1/2}$

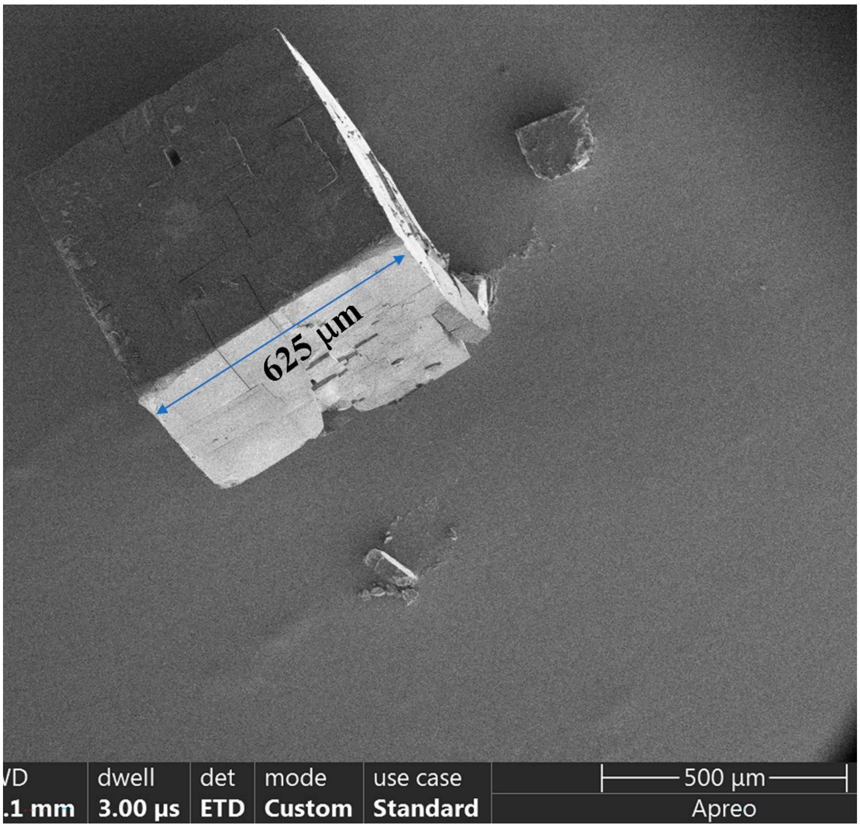

Figure S1. Scanning electron microscope (SEM) image of a single crystal of compound **1**.

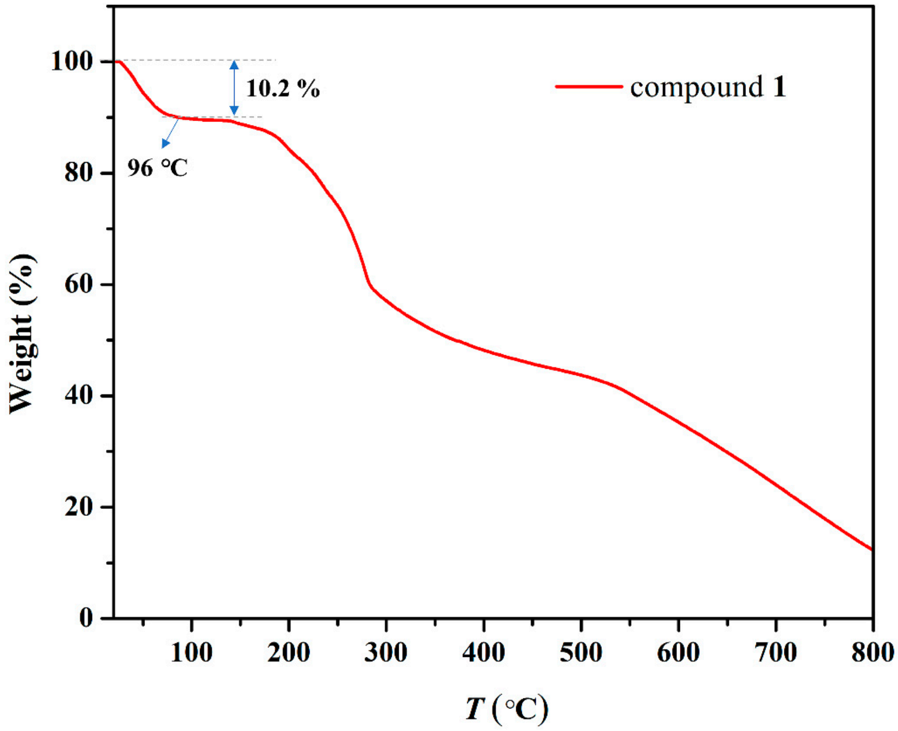

Figure S2. TGA for compound **1**.

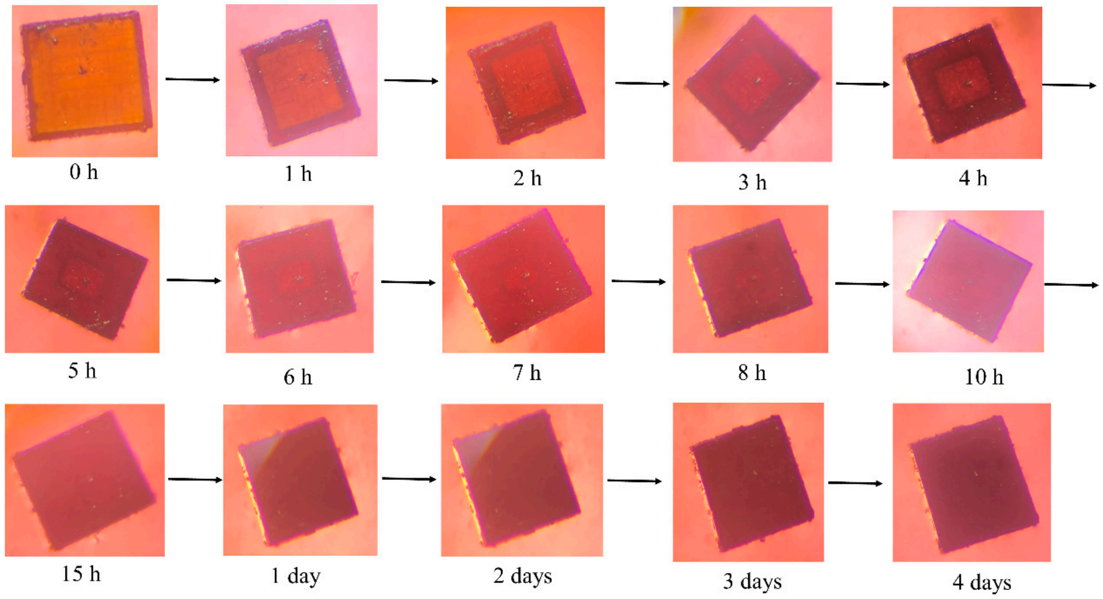

Figure S3. The photographs showing the color change of the single crystal of compound **1** at different time intervals during the metalation process.

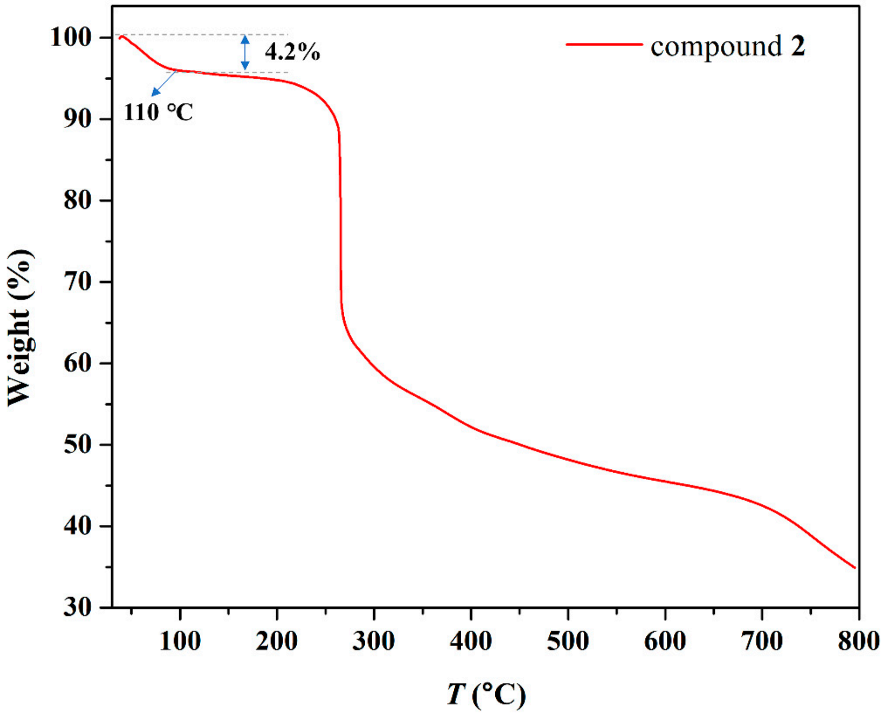

Figure S4. TGA for compound **2**.
